# Young age increases the risk of lymph-node metastasis in patients with muscle-invasive bladder urothelial carcinoma

**DOI:** 10.1186/s12885-020-07354-7

**Published:** 2020-09-04

**Authors:** Zijian Tian, Lingfeng Meng, Xin Wang, Tongxiang Diao, Maolin Hu, Miao Wang, Ming Liu, Jianye Wang

**Affiliations:** 1 Department of Urology, Beijing Hospital, National Center of Gerontology, Institute of Geriatric Medicine, Chinese Academy of Medical Sciences, Beijing, 100730 China; 2 Graduate School of Peking Union Medical College, Chinese Academy of Medical Sciences, Beijing, 100730 China

**Keywords:** Urinary bladder neoplasms, Lymph nodes, Age at diagnosis

## Abstract

**Background:**

The risk of positive lymph nodes in patients with muscle-invasive bladder urothelial carcinoma (MIBC) can be used to guide treatment recommendations. However, little is known about the effect of age on lymph node positivity (LN+). This study aimed to evaluate the effect of age on LN+ in MIBC.

**Methods:**

We analyzed patients with stage T2–T4 bladder urothelial carcinoma who had not received preoperative radiotherapy, had at least one lymph node examined, and underwent cystectomy between 1998 and 2015. The Cochran–Armitage trend test and logistic univariate and multivariate analyses were used to evaluate the effect of age on LN+ in all T stages.

**Results:**

In total, 15,624 patients with MIBC were identified, including 747 patients aged ≤50 years (4.78%), 2614 patients aged 50–59 years (16.73%), 4914 patients aged 60–69 years (31.45%), 5225 patients aged 70–79 years old (33.44%), and 2124 patients aged > 80 years (13.59%). In T2–T4 staging, LN+ was negatively correlated with age. After adjustment for several covariates, multivariate logistic regression analysis revealed that age was an independent risk factor for LN+.

**Conclusions:**

In this large SEER analysis, Young patients with MIBC have a higher risk of lymph node metastasis. This finding is worthy of further study and may eventually affect the treatment decisions of young patients.

## Background

Bladder cancer is the most common malignant tumor of the urinary system, with high morbidity and mortality. It is estimated that there will be approximately 81,400 new bladder cancer patients and 17,980 deaths due to this disease in the USA in 2020 [1]. Urothelial carcinoma accounts for approximately 90% of bladder cancers, 25% of which can develop into muscle-invasive bladder cancer [2] and approximately half of which are potentially fatal [3]. Radical cystectomy plus pelvic lymph node dissection (PLND) is considered the gold standard treatment for bladder urothelial carcinoma with **i**nvasion of the detrusor muscle [4], and up to 25% of patients turn out to have lymph node metastasis during radical cystectomy [5]. Lymph node positivity (LN+) has been shown to be an independent predictor of disease recurrence and cancer-specific death in urothelial carcinoma of the bladder [6].

In patients with muscle-invasive bladder urothelial carcinoma (MIBC), the evaluation of LN+ is very important for staging and decisions on treatment. Age is an independent predictor of LN+ in various cancers [7–9]. In an analysis of 46,077 patients with thyroid cancer, it was found that young patients were more likely to have LN+, regardless of the T stage [7]. Similarly, in patients with colon cancer, the risk of LN+ is associated with age at the time of diagnosis [8], and age is related with LN+ in non-small cell lung cancer [9].

However, the correlation between LN+ and age at the time of diagnosis in patients with MIBC is still uncertain. Therefore, in this study, we used data from the Surveillance, Epidemiology, and End Results (SEER) database and descriptive statistics to investigate the predictive effect of age on LN+ in patients with MIBC based on a large population dataset.

## Methods

### Patients

The SEER database records data on the morbidity, mortality, and illness of millions of patients with malignant tumors in certain states and counties of the USA. In this study, we analyzed the records of all patients diagnosed with MIBC between 1988 and 2015 in the SEER database. The inclusion criteria were as follows: 1. patients clinically and pathologically diagnosed as MIBC; 2. patients who underwent surgery and for whom exact pathological details were available; 3. patients who had at least one lymph node examined. The exclusion criteria were as follows: 1. MIBC patients aged < 18 years; 2. patients with distant metastasis; 3. patients who received radiotherapy and neoadjuvant chemotherapy and chemotherapy (to eliminate the effect of preoperative radiotherapy on lymph node collection and LN+); 4. patients who underwent local resection or local destruction surgery (as this type of surgery is associated with low expectation of obtaining lymph nodes).

### Data analysis

The primary outcome was LN+. Age was considered a categorical variable, and patients were classified by 10-year age groups. Considering the relatively small number of patients under the age of 50 years, we classified them all into one group. Chi-square analysis was used to compare the proportions of all classified data. The number of lymph nodes examined (LNE) was compared between the age groups by rank sum test. We used Cochran–Armitage trend tests to evaluate changes in LN+ with age. Using LN+ as the result, logistic regression univariate and multivariate analyses were performed for T2–T4 stages. Age, sex, race, tumor grade, year of operation and the number of lymph node examinations were covariates. Age ≥ 80 years was used as the reference group. Odds ratios (ORs) and 95% confidence intervals (CIs) are reported. SPSS v.25 and R v.3.6.1 were used for analyses. All statistical tests were two-sided, and *P* < 0.05 was considered significant.

## Results

### Demographic and clinicopathological characteristics

We identified 15,624 patients who met our research eligibility criteria. Table 1 summarizes the demographic and tumor characteristics. Most of the patients were aged 70–79 years (33.44%, *n* = 5225). Only 747 patients (4.78%) were aged < 50 years; 2614 (16.73%), 50–59 years; 4914 (31.45%), 60–69 years; and 2124 (13.59%), > 80 years. Grade IV (58.76%) and stage T3 (41.477%) accounted for the largest proportion in tumor grading and staging, respectively. Caucasian and afroamericans accounted for 89.15 and 5.88%, respectively. The median number of patients with stage T2 receiving LN examination was the highest (*n* = 13), and the average number of patients with stage T3 and T4 receiving LN examination was 11 and 12, respectively (Table 2).

**Table 1:** Demographic and clinicopathological characteristics by age at diagnosis

Characteristic	n (%)	Age at diagnosis, years (percent within age group)					*P*
		< 50	50–59	60–69	70–79	> 80	
**Sex**							
Male	11,886 (96.95)	574 (76.84)	2052 (78.50)	3830 (77.94)	3916 (74.95)	1514 (71.28)	
Female	3738 (3.05)	173 (23.16)	562 (21.50)	1084 (22.06)	1309 (25.05)	610 (28.72)	< 0.001
**Year of diagnosis**							
1988 to 2003	3717 (23.79)	221 (29.59)	649 (24.83)	1081 (22.00)	1323 (25.32)	443 (20.86)	
2004 to 2015	11,907 (76.21)	526 (70.41)	1965 (75.17)	3833 (78.00)	3902 (74.68)	1681 (79.14)	< 0.001
**Race**							
Caucasians	13,929 (89.15)	634 (84.87)	2260 (86.46)	4387 (89.28)	4718 (90.30)	1930 (90.87)	
Afro-Americans	918 (5.88)	73 (9.77)	220 (8.42)	308 (6.27)	238 (4.56)	79 (3.72)	
Other	751 (4.81)	37 (4.95)	128 (4.90)	211 (4.29)	264 (5.05)	111 (5.23)	
Unknown	26 (0.17)	3 (0.40)	6 (0.23)	8 (0.16)	5 (0.10)	4 (0.19)	< 0.001
**Grade**							
I	60 (0.38)	9 (1.20)	8 (0.31)	13 (0.26)	19 (0.36)	11 (0.52)	
II	469 (3.00)	28 (3.75)	96 (3.67)	139 (2.83)	152 (2.91)	54 (2.54)	
III	5388 (34.49)	272 (36.41)	907 (34.70)	1660 (33.78)	1836 (35.14)	713 (33.57)	
IV	9181 (58.76)	415 (55.56)	1512 (57.84)	2948 (59.99)	3035 (58.09)	1271 (59.84)	
Unknown	526 (3.37)	23 (3.08)	91 (3.48)	154 (3.13)	183 (3.50)	75 (3.53)	0.01
**T stage**							
T2	6353 (40.66)	331 (44.31)	1138 (43.53)	2141 (43.57)	2053 (39.29)	690 (32.49)	
T3	6479 (41.47)	281 (37.62)	1034 (39.56)	1897 (38.60)	2230 (42.68)	1037 (48.82)	
T4	2792 (17.87)	135 (18.07)	442 (16.91)	876 (17.83)	942 (18.03)	397 (18.69)	< 0.001

Characteristics differ by age group; Grade represents the degree of differentiation of bladder tumor (Grade I: Well differentiated, Grade II: Moderately differentiated, Grade III: Poorly differentiated, Grade IV: Undifferentiated); Year of diagnosis represents the time of diagnosis of MIBC; T stage represents the degree of invasion of bladder tumor

**Table 2:** Number of LNE stratified by age and T stage

Age, years	T2			T3			T4		
	n	Median LNE	25th, 75th Percentile	n	Median LNE	25th, 75th Percentile	n	Median LNE	25th, 75th Percentile
All	6353	13	7, 22	6479	11	6, 19	2792	12	6, 20
< 50	331	14	8, 23	281	15	7, 24	135	14	7, 25
50–59	1138	15	8, 25	1034	13	7, 21	442	13	6, 22.25
60–69	2141	14	7, 23	1897	12	6,21	876	12	6, 21
70–79	2053	12	6, 21	2230	10	5,18	942	11	5, 19
≥80	690	11	5, 19	1037	9	4, 17	397	10	5, 18
P^*^	< 0.001			< 0.001			< 0.001		

*from rank sum test

*LNE* lymph nodes examined

### Impact of age at diagnosis on LN+

To explore the effect of age on LN+, we grouped patients according to T stage. In total, 4321 patients (27.66%) had lymph node metastasis. The rate of LN+ was 12.37% in T2, 34.68% in T3, and 46.13% in T4. In all stages, age was significantly negatively correlated with LN+ (*P* < 0.05; Table 3).

**Table 3:** LN+ rate and age within T-stage groups

Age,	T2		T3		T4	
years	All patients	LN+ (%)	All patients	LN+ (%)	All patients	LN+ (%)
All	6353	786 (12.37)	6479	2247 (34.68)	2792	1288 (46.13)
< 50	331	52 (15.71)	281	112 (39.86)	135	71 (52.59)
50–59	1138	156 (13.71)	1034	418 (40.43)	442	228 (51.58)
60–69	2141	281 (13.12)	1897	710 (37.43)	876	406 (46.35)
70–79	2053	232 (11.30)	2230	670 (30.04)	942	411 (43.63)
≥80	690	65 (9.42)	1037	337 (32.50)	397	172 (43.32)
P^*^	0.01		< 0.001		0.03	

*from Cochran–Armitage trend test

*LN+* lymph node positivity

### Multivariable analysis of LN+

We used multivariate logistic regression to evaluate whether the association between age and LN+ at the time of diagnosis was independent of other known risk factors. Sex, race, year of operation, tumor grade, and LNE were used as covariates of the adjusted model. Age remained a significant predictor of LN+ in all T stages (Table 4, *P* < 0.05), and young MIBC patients were more likely to have LN+. Compared with the reference group (aged > 80 years), patients with T2 and T4 stages, aged < 50 years, were more likely to have LN+, and the adjusted OR was 1.805 (95% CI, 1.217–2.676) and 1.492 (95% CI, 1.002–2.220), respectively. The probability of LN+ was the highest in the 50–59-year age group with T3, and the adjusted OR was 1.407 (95% CI, 1.173–1.688).

**Table 4:** Association of age and rate of LN+

Age	T2		T3		T4	
years	Unadjusted	Adjusted for covariates	Unadjusted	Adjusted for covariates	Unadjusted	Adjusted for covariates
< 50	1.792 (1.212–2.650)	1.805 (1.217–2.676)	1.377 (1.049–1.807)	1.360 (1.033–1.790)	1.451 (0.981–2.147)	1.492 (1.002–2.220)
50–59	1.527 (1.125–2.075)	1.551 (1.139–2.111)	1.409 (1.178–1.687)	1.407 (1.173–1.688)	1.394 (1.061–1.830)	1.411 (1.070–1.861)
60–69	1.453 (1.093–1.931)	1.467 (1.102–1.952)	1.242 (1.059–1.458)	1.237 (1.053–1.453)	1.130 (0.890–1.435)	1.146 (0.900–1.459)
70–79	1.225 (0.917–1.637)	1.239 (0.926–1.656)	0.892 (0.761–1.045)	0.897 (0.765–1.052)	1.013 (0.799–1.283)	1.020 (.803–1.296)
≥80 (reference)	1.000	1.000	1.000	1.000	1.000	1.000
P^*^	0.007	0.007	< 0.001	< 0.001	0.025	0.022

*All statistical tests were two sided

*LN+* lymph node positivity

## Discussion

Tumor stage and grade are associated with the risk of lymph node metastasis in MIBC [10, 11]. However, it is not ideal to use staging and grading alone to predict lymph node metastasis. The addition of other important prediction factors, such as age, may improve the risk stratification of patients, and more active, multi-modal treatment may be selected for high-risk patients, thus improving the prognosis. No study has explored the predictive effect of age on LN+ in patients with MIBC. In the current study, we analyzed data from 15,624 MIBC patients extracted from the SEER database. We found that young patients had a higher tendency for LN+ at any T stage. This finding was validated in multivariate analysis including sex, race, grade, LNE, and year of operation. This is consistent with the results reported by Hellenthal et al. that per 10-year age increase, the odds of LN+ in patients with bladder cancer decreased by approximately 20% [12].

The effect of age on LN+ may be related to biological differences between young and old patients. Migaldi et al. pointed out that low p27Kip1 expression was not related to the risk of recurrence in young patients, whereas decreased p27Kip1 expression was related to an increased risk of recurrence in older patients [13]. More significantly, high Ki67 expression and low cyclinD1 expression were associated with an increased risk of recurrence in young patients, but not in older patients. Thus, compared with older patients, urothelial carcinoma of the bladder in young patients may involve different molecular pathways. In addition, with increasing age, various changes occur in the body, including those in the lymph nodes [14]. Aging leads to a decrease in the cortex and medulla of the lymph nodes and an increase in degeneration into inactive lymph nodes without lymph node tissue, resulting in reduced lymph flow to and retraction of the lymph nodes [15, 16]. This may be one of the important reasons for the effect of age on LN+.

Lymph node dissection is an indispensable part of radical resection of bladder cancer. The reasonable PLND has been proved to be helpful in determing the pathological stage, guide follow-up treatment [5]. However, it is still controversial whether PLND can improve the prognosis of patients. In a retrospective study, young patients with radical cystectomy and PLND had better all cause and cancer specific survival than radical cystectomy alone [17]. Another retrospective study have suggested that the survival prognosis of extended lymphadenectomy group is better than that of standard lymphadenectomy group [18]. But it’s worth noting that, there is a great risk of Will Rogers Phenomena and Stage migration, so the whole concept is blurred on a scientific level when examining retrospective materials [19]. Choi et al. found that compared with standard lymphadenectomy, super-extended or extended lymphadenectomy may have no significant effect on local recurrence, distant metastasis, disease-specific survival and overall survival [20]. To date, the only randomized phase 3 trial showed no improvement in PLND in either recurrence-free survival, cancer-specific survival or overall survival [21]. Therefore, more randomized prospective trials are needed to establish whether PLND can benefit patients with radical resection of bladder cancer. Moreover, extended lymphadenectomy can increase the time of operation and cause potential bleeding, lymphatic leakage, lymphoceles, autonomic nerve and ureteral injury, and serious nutritional and immune problems after operation, which significantly prolong the risk of postoperative rehabilitation and hospitalization [21–25]. This refutes the hypothesis that the benefits associated with PLND are consistent across ages and comorbidities. Considering the lack of a final conclusion on the scope and benefits of lymph node dissection, based on current research results, in elderly patients, because the probability of LN+ is low and their physique is generally weaker, lymph node involvement should be actively evaluated before operation. In line with our results, Koppie et al. found that for elderly patients with bladder cancer or with more underlying diseases, LN+ was lower with PLND, and thus, they recommended that PLND or regional lymph node dissection should not be performed [26]. Another study also pointed out that in older and seriously ill patients, radical cystectomy combined with PLND had no significant clinical benefit compared with radical cystectomy alone [17]. When choosing the specific way of lymph node dissection for the elderly, we should try our best to consider the time of operation and anesthesia, and the possible impact of cardiopulmonary complications on the elderly.

Clinical treatment strategies for patients with MIBC vary according to the status of lymph nodes. Accurate prediction of lymph node metastasis is essential to help doctors make reasonable decisions, especially for patients who need to be evaluated for lymph node status before surgery or do not need PLND. Trimodality therapy (TMT) is an alternative for patients who do not undergo or refuse radical resection of bladder cancer [27]. While preserving bladder function, TMT improves the long-term survival rate and the quality of life similar to radical resection of bladder cancer [28–30]. However, TMT is not recommended when patients show high-risk features, such as LN+ [27, 31]. Therefore, for patients who opt for TMT, it is important to carefully evaluate lymph node involvement before operation. Because the survival time of young patients with bladder cancer is longer [32], and according to our results, young patients are more prone to LN+, a comprehensive and professional evaluation of the lymph node area should be done before deciding on TMT, so as to avoid missed diagnosis of lymph node involvement and wrong treatment.

Several limitations of our study should be noted. First, this study is limited by its long term and retrospective nature. The way patients receive surgery is affected by the year of diagnosis and clinical factors. These factors were taken into account in the multivariate analysis. However, the clinical treatment of young patients may be more aggressive than that of older patients, which leads to systematic bias. Second, the SEER database collects data from a large number of patients from the population-based cancer registry, but some data may be miscoded or omitted during the registration process. However, this error coding is random and does not introduce any system bias. Finally, the data are representative only for the population in the SEER area and do not apply to other geographic locations.

## Conclusions

Our analysis of data in the SEER database showed that after considering other predictive factors, there is an increased risk of LN+ in young MIBC patients. Considering the different lymph node invasiveness in patients of different ages, our results can guide clinicians to choose the best treatment. Our findings are worthy of further study and may influence the assessment of lymph node invasiveness in patients with MIBC.

## Data Availability

In this retrospective study, our data were downloaded from the SEER database.

## Abbreviations

LN+: Lymph node positivity
LNE: Lymph nodes examined
MIBC: Muscle-invasive bladder urothelial carcinoma
NAC: Neoadjuvant chemotherapy
PLND: Pelvic lymph node dissection
SE: Standard error
SEER: Surveillance Epidemiology and End Results
TMT: Trimodality therapy
